# Prognostic Value of Coronary Computed Tomography Angiography–Derived Fractional Flow Reserve From a Large Nationwide Cohort

**DOI:** 10.1161/CIRCULATIONAHA.126.081264

**Published:** 2026-09-22

**Authors:** Jack S. Bell, Edward Nicol, Vinod Venugopal, Liam Mullen, Laurence Tidbury, Matthew Shaw, Campbell Rogers, Sarah Mullen, Bjarne Linde Nørgaard, Jonathon Leipsic, Jonathan R. Weir-McCall, Timothy A. Fairbairn, on behalf of the FISH&CHIPS Investigators, Sumeet Sharma

**Affiliations:** Blackpool Teaching Hospitals NHS Foundation Trusts; Dudley Group NHS Foundation Trust; Frimley Health NHS Foundation Trust; Great Western Hospitals NHS Foundation Trust; Guys and St Thomas’ NHS Trust; Hull University Teaching Hospitals NHS Trust; Leeds Teaching Hospitals NHS Trust; Liverpool Heart and Chest Hospital NHS Foundation Trust; Manchester University NHS Foundation Trust; Newcastle Upon Tyne Hospitals NHS Foundation Trust; North Tees and Hartlepool NHS Foundation Trust; Portsmouth Hospitals NHS Trust; Royal Devon and Exeter Hospital NHS Trust; Royal Papworth Hospital NHS Foundation Trust; Royal United Hospitals Bath NHS Foundation Trust; Sheffield Teaching Hospitals NHS Foundation Trust; United Lincolnshire Hospitals NHS Trust; University Hospitals Bristol NHS Foundation Trust; University Hospital Dorset NHS Trust; University Hospitals of Leicester NHS Trust; University Hospitals of North Midlands NHS Trust; University Hospitals Sussex NHS Foundation Trust; West Hertfordshire Hospital NHS Trust; 1 Department of Cardiology, Liverpool Heart and Chest Hospital, Liverpool, United Kingdom (J.S.B., L.M., T.A.F.).; 2 Liverpool Centre for Cardiovascular Science at the University of Liverpool, Liverpool, United Kingdom (J.S.B., L.T., M.S., T.A.F.).; 3 School of Biomedical Engineering and Imaging Sciences, King’s College London, London, United Kingdom (E.N., J.R.W.-M.).; 4 Departments of Cardiology and Radiology, Royal Brompton Hospital, Guys and St. Thomas’ NHS Foundation Trust, London, United Kingdom (E.N., J.R.W.-M.).; 5 United Lincolnshire Hospitals, Lincoln, United Kingdom (V.V.).; 6 Heartflow Inc 479990 , Mountainview, CA (C.R., S.M.).; 7 Institute of Clinical Medicine, Aarhus University, Aarhus, Denmark (B.L.N.).; 8 Department of Cardiology, Aarhus University Hospital, Aarhus, Denmark (B.L.N.).; 9 Centre for Cardiovascular Innovation and Center for Heart Valve Innovation, St. Paul’s and Vancouver General Hospital, Division of Cardiology and Department of Radiology, University of British Columbia, Vancouver, BC, Canada (J.L.).

**Keywords:** coronary computed tomography angiography, fractional flow reserve, myocardial infarction, prognosis

## Abstract

**BACKGROUND:**

The diagnostic value of coronary computed tomography angiography–derived fractional flow reserve (FFR-CT) in stable coronary artery disease is well established. The prognostic value of predicting future cardiovascular events is less clear, as studies are limited by small populations, variable measurement points, composite outcomes, and short follow-up.

**OBJECTIVES:**

A nationwide cohort of coronary computed tomography angiography with 3-year follow-up was used to examine the association between FFR-CT measurements and future cardiovascular events.

**METHODS:**

Patients receiving coronary computed tomography angiography and clinically indicated FFR-CT analysis (HeartFlow) for stable coronary artery disease between 2017 and 2020 at 27 sites across England were included. Lowest per-patient stenosis-specific and distal vessel FFR-CT measurements were related to individual outcomes of myocardial infarction (MI), cardiovascular mortality, all-cause mortality, and revascularization. FFR-CT measurements were divided into subgroups to investigate for a gradient of risk: normal FFR-CT (>0.8), borderline (0.71–0.8), reduced (0.51–0.7), and severely reduced (≤0.5).

**RESULTS:**

In total, 7836 patients aged 63 (56.0–71.0), 37.4% women, underwent FFR-CT analysis. Stenosis-specific FFR-CT was associated with future MI (hazard ratio [HR], 2.08–5.73; *P*<0.001), cardiovascular death (HR, 1.51–3.40; *P*<0.001), all-cause death (HR, 1.01–1.58; *P*=0.01), and revascularization (HR, 4.79–24.48; *P*<0.001), with lower FFR-CT values associated with incrementally greater risk. After adjusting for cardiovascular risk factors and coronary stenosis severity (Coronary Artery Disease Reporting and Data System), stenosis-specific FFR-CT≤0.7 was associated with MI (adjusted HR, 2.94–3.39; *P*<0.001), ≤0.5 with cardiovascular death (adjusted HR, 2.92; *P*=0.02), all-cause death (adjusted HR, 1.49; *P*=0.045), and ≤0.8 with revascularization (adjusted HR, 3.02–9.42; *P*<0.001). The addition of FFR-CT improved discrimination for MI or cardiovascular death compared with a risk factor and stenosis severity model (C index, 0.783; ∆C index, 0.012; *P*<0.001). An FFR-CT value of 0.67 defined the optimal cutoff for risk of future cardiovascular events. Distal-vessel FFR-CT was associated with outcomes similar to stenosis-specific FFR-CT. Stenosis-specific and distal-vessel FFR-CT discordance was common (36.4%), but when stenosis-specific FFR-CT was normal, event rates were low (1.5% at 3 years for MI or cardiovascular death).

**CONCLUSIONS:**

FFR-CT is associated with the individual outcomes of MI, cardiovascular death, and all-cause death independent of risk factors and stenosis severity, with lower FFR-CT values associated with worse outcomes. Reduced and severely reduced FFR-CT have the strongest association with future cardiovascular events, with a value of 0.67 defining the optimal risk threshold.

**Clinical Perspective**

**What Is New?**

- Coronary computed tomography angiography fractional flow reserve (FFR-CT) predicts the individual outcomes of myocardial infarction (MI), cardiovascular death, all-cause death, and revascularization independent of clinical risk factors and coronary artery stenosis severity.
- There is a continuum of risk with decreasing FFR-CT, and a binary stenosis-specific threshold of 0.67 identifies individuals at increased risk for future MI and cardiovascular death.
- By directly comparing stenosis-specific and distal-vessel FFR-CT, we show similar discrimination for future cardiovascular events, with stenosis-specific FFR-CT having a stronger association with MI and cardiovascular death and distal-vessel FFR-CT with all-cause death.

**What Are the Clinical Implications?**

- FFR-CT is additive to clinical risk factors and stenosis severity in discriminating future MI and cardiovascular death and reclassifying risk.
- Borderline “gray zone” FFR-CT (0.71–0.80) has a weak association with future MI, while FFR-CT≤0.70 is associated with a robust increase in risk for both MI and cardiovascular death, identifying high-risk patients who may benefit most from preventive therapy.
- Stenosis-specific and distal-vessel FFR-CT discordance is frequent, but major discordance (>0.80 versus <0.71) is rare; thus, it is reasonable to focus on stenosis-specific FFR-CT for both diagnosis and prognosis of flow-limiting coronary artery disease.

Coronary computed tomography angiography (CCTA) is a first-line investigation for suspected stable coronary artery disease (CAD) with excellent sensitivity for detecting coronary plaque. Historically, CCTA was limited in relating a coronary stenosis to flow reduction, and therefore angina symptoms, often requiring downstream functional investigations or invasive coronary angiography. This limitation has been addressed with CCTA-derived fractional flow reserve (FFR-CT), which uses principles of computational fluid dynamics to estimate flow in the coronary tree. FFR-CT has been validated against invasive FFR,^1–3^ and trials have shown that a diagnostic strategy incorporating FFR-CT reduces the rate of unnecessary invasive coronary angiography at a neutral cost.^4–6^ Therefore, FFR-CT is recommended for use in the chest pain pathway as a diagnostic aid to CCTA.^7^

Akin to invasive FFR, FFR-CT may provide prognostic information for predicting future major adverse cardiovascular events.^8,9^ However, the evidence to date is based on small numbers, composite outcomes, or a limited follow-up period. It therefore remains uncertain whether FFR-CT offers incremental prognostic value beyond traditional risk factors and stenosis severity. This question is particularly relevant given the ongoing debate over whether myocardial ischemia is itself prognostic or simply a downstream marker of anatomical CAD severity.^10,11^

Interpreting the FFR-CT evidence to date is further compounded by differing measurement approaches used across studies. Stenosis-specific FFR-CT (2 cm distal to a stenosis) is recommended for clinical use, as it has the highest correlation with lesion-specific ischemia and increases specificity for identifying flow-limiting coronary stenoses.^12,13^ The relevance of distal-vessel FFR-CT values has been questioned, as they are frequently “positive” in the instance of mild coronary disease and may lead to unnecessary invasive testing if interpreted incorrectly. However, distal values may better reflect diffuse coronary disease that may be clinically relevant.^14^ It remains to be answered whether distal values are prognostically important and should be incorporated when reporting FFR-CT.

To address these uncertainties, this study used a large nationwide cohort with sufficient clinical events and follow-up to determine the association between FFR-CT and individual cardiovascular outcomes, which FFR-CT measurement has the greatest prognostic value, and whether these variables add incremental prognostic information beyond traditional cardiovascular risk factors and stenosis severity.

## METHODS

### Study Population

All patients receiving FFR-CT from the FISH&CHIPS (FFR-CT in Stable Heart Disease and Coronary Computed Tomography Angiography Helps/Hinders Improved Patient Care and Societal Costs) study were included. FISH&CHIPS was a multicenter, retrospective, quasi-experimental observational cohort study of 90 553 patients, investigating the impact of a national FFR-CT implementation program on patient cardiovascular outcomes and cardiac tests. The study design has been reported previously.^6^ In brief, all patients ≥18 years, investigated with CCTA for suspected CAD from 27 English hospitals between 2017 and 2020, were included. The decision to send a CCTA for FFR-CT analysis was at the discretion of the clinical team. Clinical management was informed following the availability of the FFR-CT result via a written report and online access to the patient-specific anatomical and physiological 3D reconstruction.^6^

### FFR-CT Analysis

FFR-CT analysis (HeartFlow Inc, Mountain View, CA) reported distal FFR-CT in the main epicardial vessels and stenosis-specific FFR-CT 2 cm distal to a stenosis ≥30% in any vessel ≥1.8 mm. When a stenosis-specific FFR-CT value was in the distal segment of the vessel, it was used as both the distal and stenosis-specific result. Patients were categorized on the basis of the lowest FFR-CT value: ≤0.50 (severely reduced), 0.51 to 0.70 (reduced), 0.71 to 0.80 (borderline), and >0.80 (normal). In the instance of multivessel CAD or sequential stenoses, the lowest per-patient FFR-CT values were used. Total occlusions were categorized as FFR-CT ≤0.50. Stenosis-specific FFR-CT values were categorized as not available when there was no stenosis ≥30%.

### Data Collection

The clinical outcomes of all-cause death, cardiovascular death, myocardial infarction (MI), and revascularization were collected from the National Health Service’s digital hospital episode statistics health care data using a previously validated methodology.^6,15,16^ Diagnostic codes from the *Classification of Interventions and Procedures, Fourth Revision*, and *International* *Classification of Diseases, Tenth* *Revision*, and World Health Organization were used to define clinical events: MI (I21/I22), percutaneous intervention (K69 and K70), and coronary artery bypass grafting (K40–K46). The office for national statistics reported all-cause and cardiovascular deaths (I00–I99). Medication data were taken from the Business Service Authority using prescriptions from the 2 years following the index CCTA.

### Ethics

The Confidentiality Advisory Group approved the use of patient data without consent based on health and social care research in the public interest (National Health Service Act 2006, s251, “Control of Patient Information”). Ethics approval was obtained from the Health Regulatory Authority, and the study was performed in accordance with the principles of good clinical practice.

### Statistical Analysis

Continuous variables are reported as mean (±SD) or median (interquartile range) and categorical as number (percent). Between-group differences were assessed by ANOVA for continuous variable and a Cochran-Armitage trend test for categorical differences, with the “no stenosis-specific value” group representing the category with the least disease. Time to first event was calculated using Kaplan-Meier methodology from the time of the CCTA. Cox proportional regression was used to determine the univariable hazard ratio (HR) for individual clinical outcomes post FFR-CT, and the proportional hazard assumptions were tested using Shoenfeld residuals. Fine-Grey modeling was performed to account for competing risks where necessary. Multivariable Cox regression models were used to adjust for patient cardiovascular risk factors (age, sex, hypertension, diabetes, dyslipidemia) and the Coronary Artery Disease Reporting and Data System (CAD-RADS) score. Models were developed to determine the performance of baseline risk factors and demographics (model 1), +CAD-RADS score (model 2), +FFR-CT (model 3), and +delta FFR-CT (model 4) for the combined end point of MI and cardiovascular death. Model performance was assessed using receiver operating characteristic analysis with Harrell’s C statistic and the Akaike information criterion with the Brier score as a measure of model calibration. FFR-CT was entered into an adjusted multivariable model as a continuous variable to provide an adjusted HR (aHR) per 0.01 unit decrease in stenosis-specific FFR-CT. A restricted cubic spline curve analysis with 3 knots was performed to assess the relationship of FFR-CT as a continuous variable to the clinical outcomes. The 95% CIs are reported, and 2-sided *P*<0.05 is considered statistically significant. All statistical analyses were performed in the R stats package (R documentation, version 3.6.2).

### Data Availability

Deidentified participant data that underlie the study reported outcomes will be made available for research collaborations upon request following review and approval by the sponsor’s director of research in accordance with Confidentiality Advisory Group approval and NHS digital data sharing agreement.

## RESULTS

### Study Population and Clinical Outcomes

Of 90 553 patients receiving CCTA, 8532 cases were submitted for FFR-CT analysis, and 7836 patients completed FFR-CT analysis (Figure 1); 7084 patients had a stenosis-specific FFR-CT reported, and 7836 patients had a distal FFR-CT reported. The median age was 63 (interquartile range, 56.0–71.0), 37.4% were women with multiple cardiovascular risk factors (16.9% diabetes, 52% hypertension, and 35.4% dyslipidemia). Cardiovascular risk factors were more prevalent as the FFR-CT decreased. High levels of medication were used (statin, 82%; aspirin, 64%) and were more frequently prescribed as FFR-CT reduced. Clinical outcomes reported at a median follow up of 3.1 years (interquartile range, 2.8–3.5 years) were 191 MIs (2.4%), 74 cardiovascular deaths (0.9%), 261 all-cause deaths (3.3%), and 1573 revascularizations (20.1%) (Table 1).

**Table 1.**
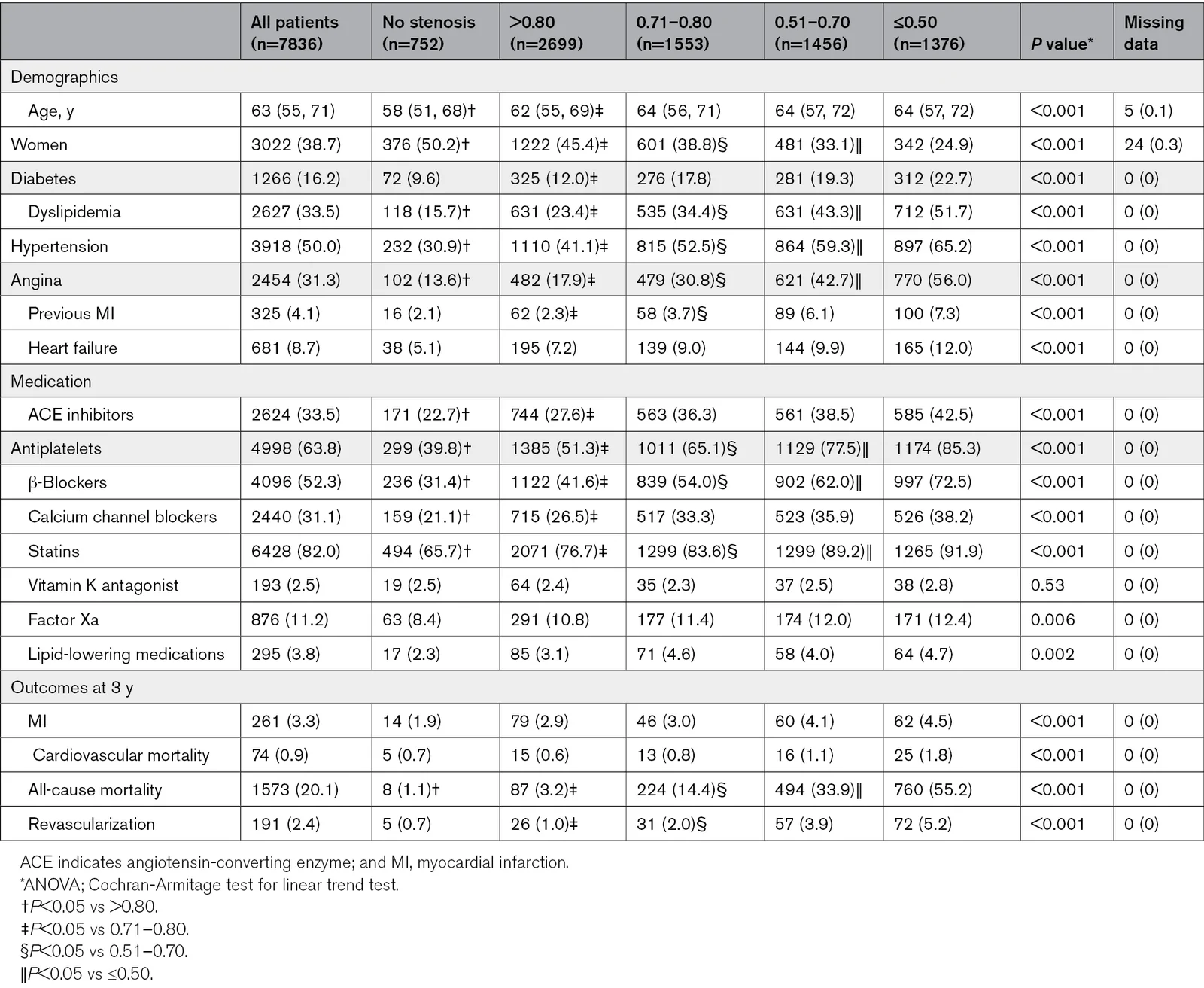
Patient Characteristics Stratified by Stenosis-Specific FFR-CT Value

**Figure 1.**
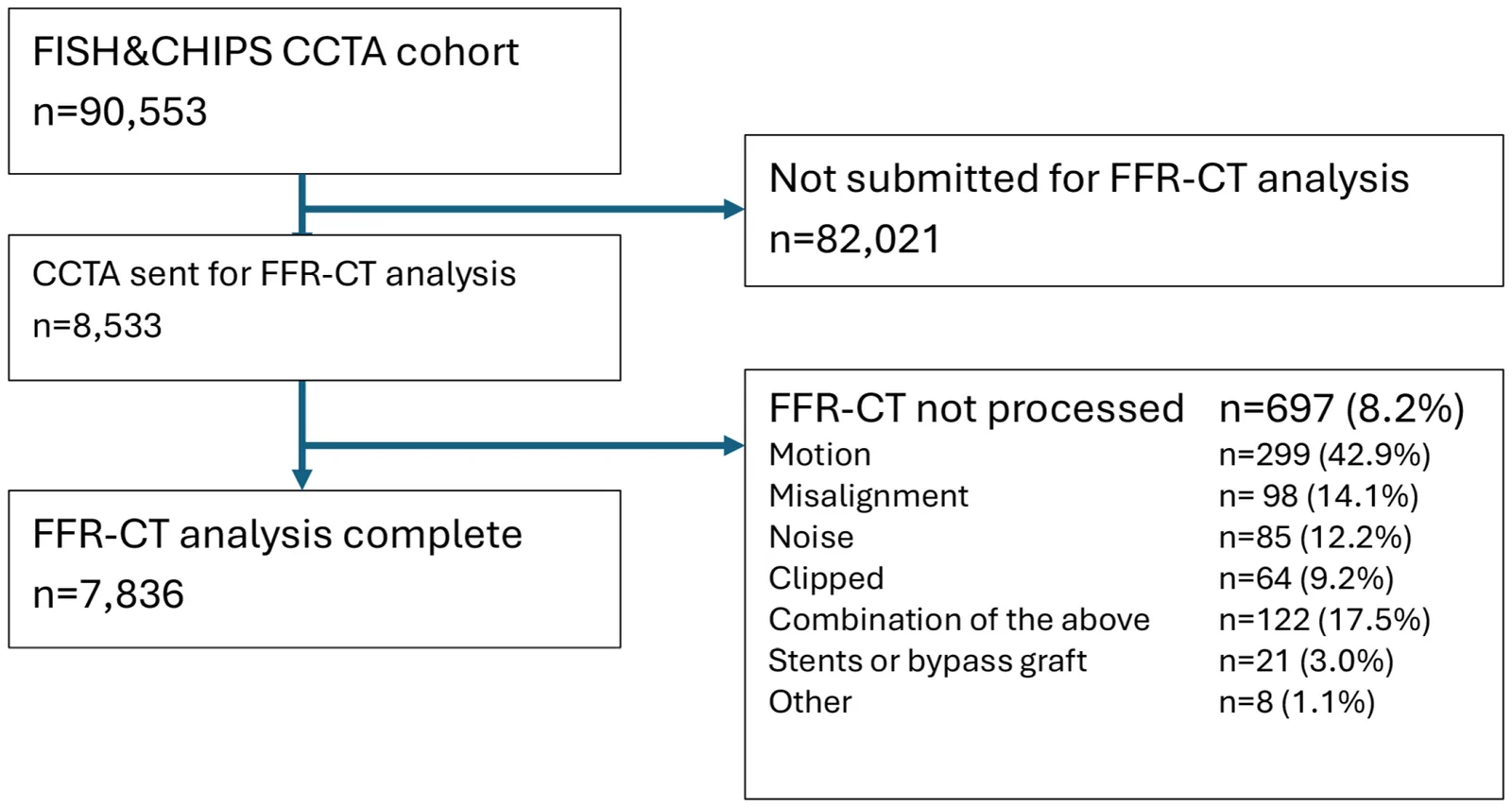
**Study flow diagram showing reasons for exclusion.** CCTA indicates coronary computed tomography angiography and FISH&CHIPS, FFR-CT in Stable Heart Disease and Coronary Computed Tomography Angiography Helps/Hinders Improved Patient Care and Societal Costs.

### Stenosis-Specific FFR-CT

Event rates were higher as stenosis-specific FFR-CT decreased (Table 1). Stenosis-specific FFR-CT categories stratified risk of MI (*P*<0.001), cardiovascular mortality (*P*<0.001), all-cause mortality (*P*=0.002), and revascularization (*P*<0.001), with lower FFR-CT values associated with higher event rates (Figure 2). All stenosis-specific FFR-CT subgroups ≤0.80 were associated with future MI and revascularization, while only the reduced (0.51–0.70) and severely reduced (≤0.50) subgroups were associated with cardiovascular mortality and all-cause mortality (Table S1). Following multivariable adjustment for clinical risk factors (age, sex, hypertension, diabetes, dyslipidemia) and CAD severity (CAD-RADS score), all FFR-CT subgroups ≤0.80 were independently associated with an increased risk for revascularization, with reduced and severely reduced FFR-CT remaining associated with MI (Table 2). Severely reduced FFR-CT was associated with cardiovascular mortality, and reduced FFR-CT was associated with all-cause mortality (Table 2). These relationships were consistent on sensitivity analysis with exclusion of occluded vessels (Table S2).

**Table 2.**
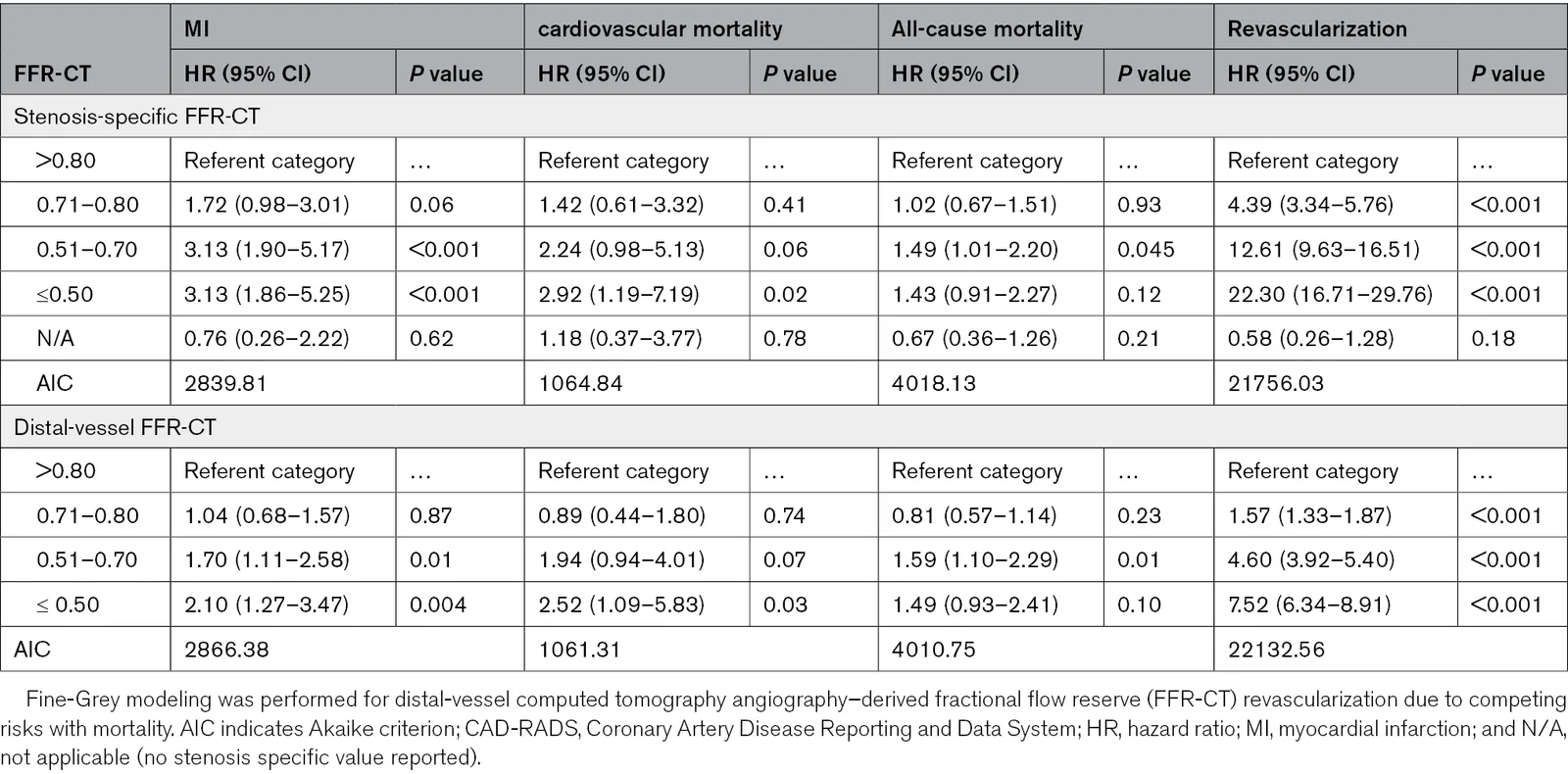
Multivariable Cox Regression for Association Between FFR-CT Subgroups and Cardiovascular Outcomes, Adjusted for Age, Sex, Hypertension, Diabetes, Dyslipidemia, and CAD-RADS Category

**Figure 2.**
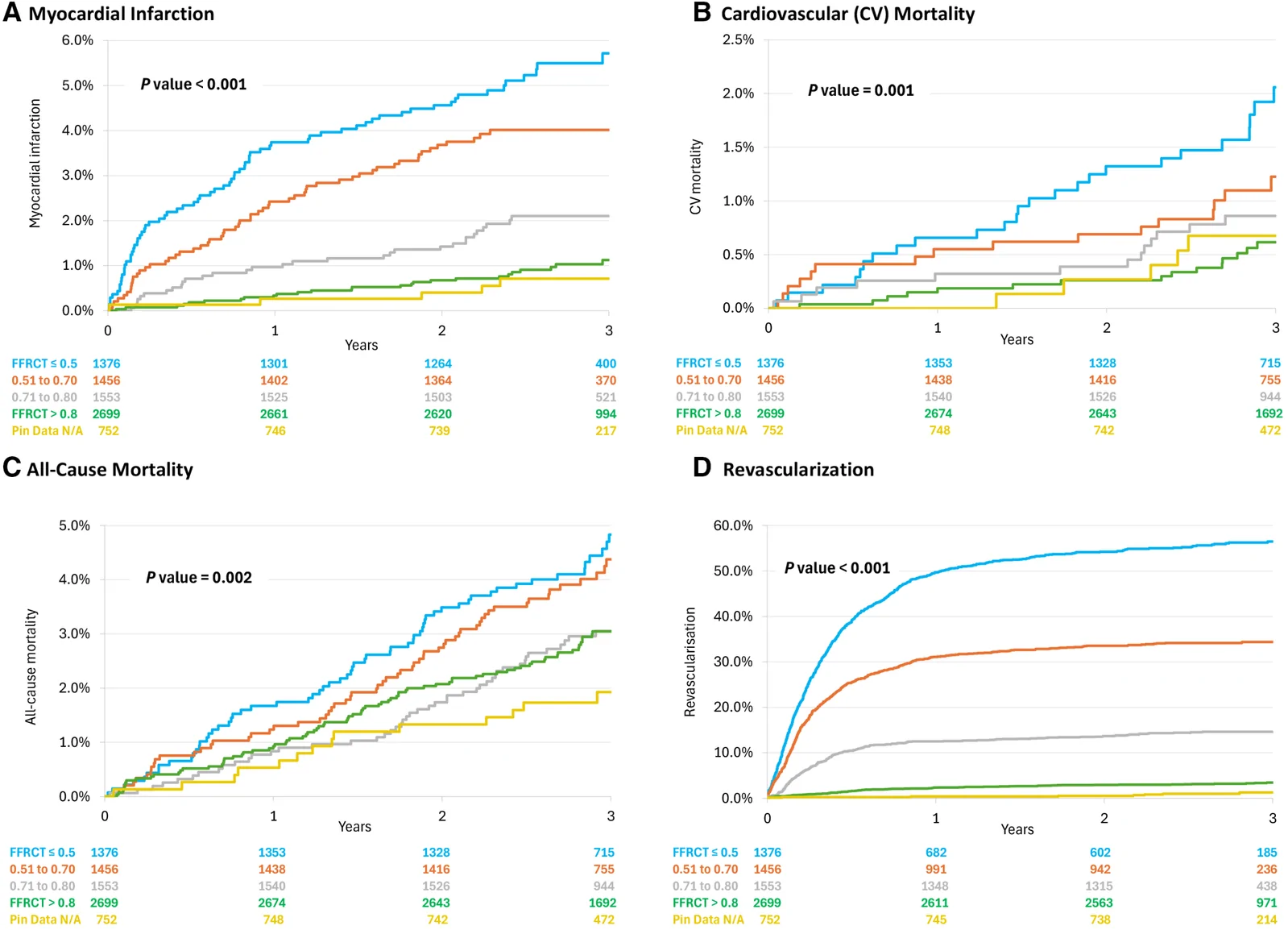
**Stenosis-specific computed tomography angiography–derived fractional flow reserve (FFR-CT) subgroups and outcomes.** Shown are Kaplan-Meier curves for individual outcomes grouped according to stenosis-specific FFR-CT values. Log-rank *P* values are shown. N/A indicates not applicable (no stenosis specific value reported).

### Incremental Predictive Value Over Established Risk Factors

The addition of coronary stenosis severity (CAD-RADS) to a model containing clinical risk factors (age, sex, hypertension, diabetes, dyslipidemia) showed improved discrimination for nonfatal MI or cardiovascular death (C index, 0.771 versus 0.758; *P*=0.007). Including stenosis-specific FFR-CT further improved discrimination (C index, 0.783 versus 0.771; *P*<0.001) compared with the clinical risk factor and CAD-RADS model (Figure 3). Addition of delta FFR-CT (**≥**0.12 cutoff) did not improve discrimination (C index, 0.782 versus 0.783; *P*=0.72) (Figure 3).

**Figure 3.**
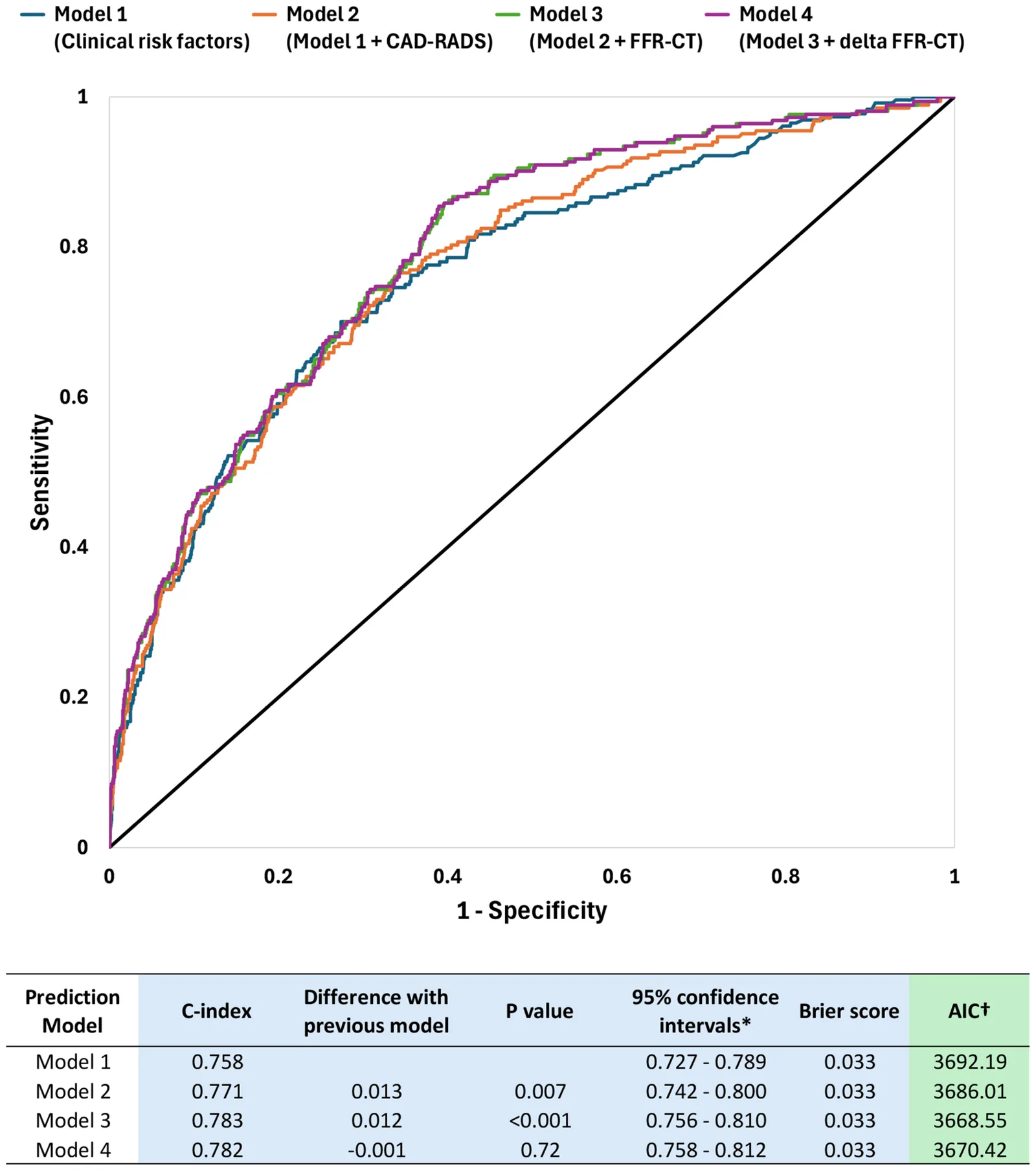
**Comparison of predictive models for a composite end point of nonfatal myocardial infarction and cardiovascular death.** Receiver operating curve plots for models predicting nonfatal myocardial infarction and cardiovascular death. Model 1: age, sex, hypertension, diabetes, hypercholesterolemia. Model 2: model 1+Coronary Artery Disease Reporting and Data System (CAD-RADS) score. Model 3: model 2+lowest stenosis-specific computed tomography angiography–derived fractional flow reserve (FFR-CT). Model 4: model 3+delta FFR-CT (≥0.12 as the threshold). AIC indicates Akaike criterion.

### FFR-CT as a Continuum for Predicting Future Events

Entering stenosis-specific FFR-CT as a continuous variable into a multivariable model adjusted for clinical risk factors and CAD-RADS showed a significant relationship to MI (aHR, 1.04 per 0.1-unit decrease [95% CI, 1.01–1.15]; *P*<0.001), cardiovascular mortality (aHR, 1.08 [95% CI, 1.008–1.73]; *P*=0.03), and revascularization (aHR, 1.006 [95% CI, 1.003–1.009]; *P*<0.001) but not for all-cause mortality (aHR, 1.50 [95% CI, 0.16–1.56]; *P*=0.23). Restricted cubic spline analysis of stenosis-specific FFR-CT as a continuous variable identified an optimal stenosis-specific FFR-CT cutoff for nonfatal MI or cardiovascular death of 0.67 (Figure 4).

**Figure 4.**
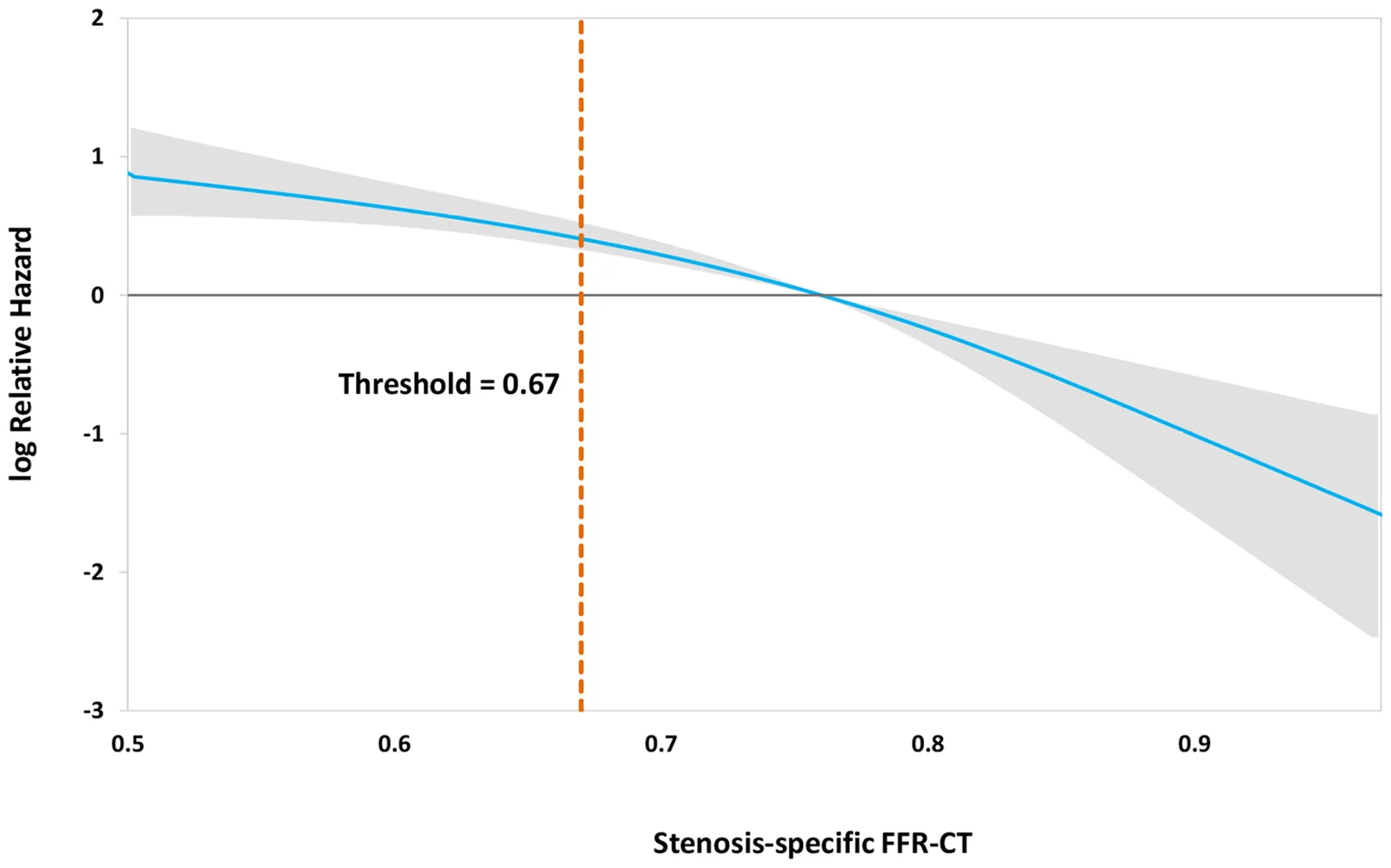
**Restricted cubic splines fitted to a Cox proportional hazards model estimating hazards for myocardial infarction or cardiovascular mortality 3 years post coronary computed tomography angiography.** Shown are restricted cubic splines with 3 knots indicating a cutoff point at stenosis-specific computed tomography angiography–derived fractional flow reserve (FFR-CT).

### Distal-Vessel FFR-CT

Distal-vessel FFR-CT categories also incrementally stratified risk of MI, cardiovascular mortality, all-cause mortality, and revascularization (all *P*<0.001) (Figure 5). All distal FFR-CT subgroups ≤0.80 were associated with revascularization, but only the reduced and severely reduced subgroups were associated with MI, cardiovascular mortality, and all-cause mortality (Table S1). Following multivariable adjustment for clinical risk factors and CAD-RADS score, severely reduced FFR-CT remained significantly associated with future MI and cardiovascular mortality, where reduced FFR-CT was associated with all-cause mortality (Table 2). The average distal-vessel FFR-CT across the 3 main epicardial coronaries had no relationship to MI or cardiovascular death but was associated with revascularization and all-cause mortality (Table S3).

**Figure 5.**
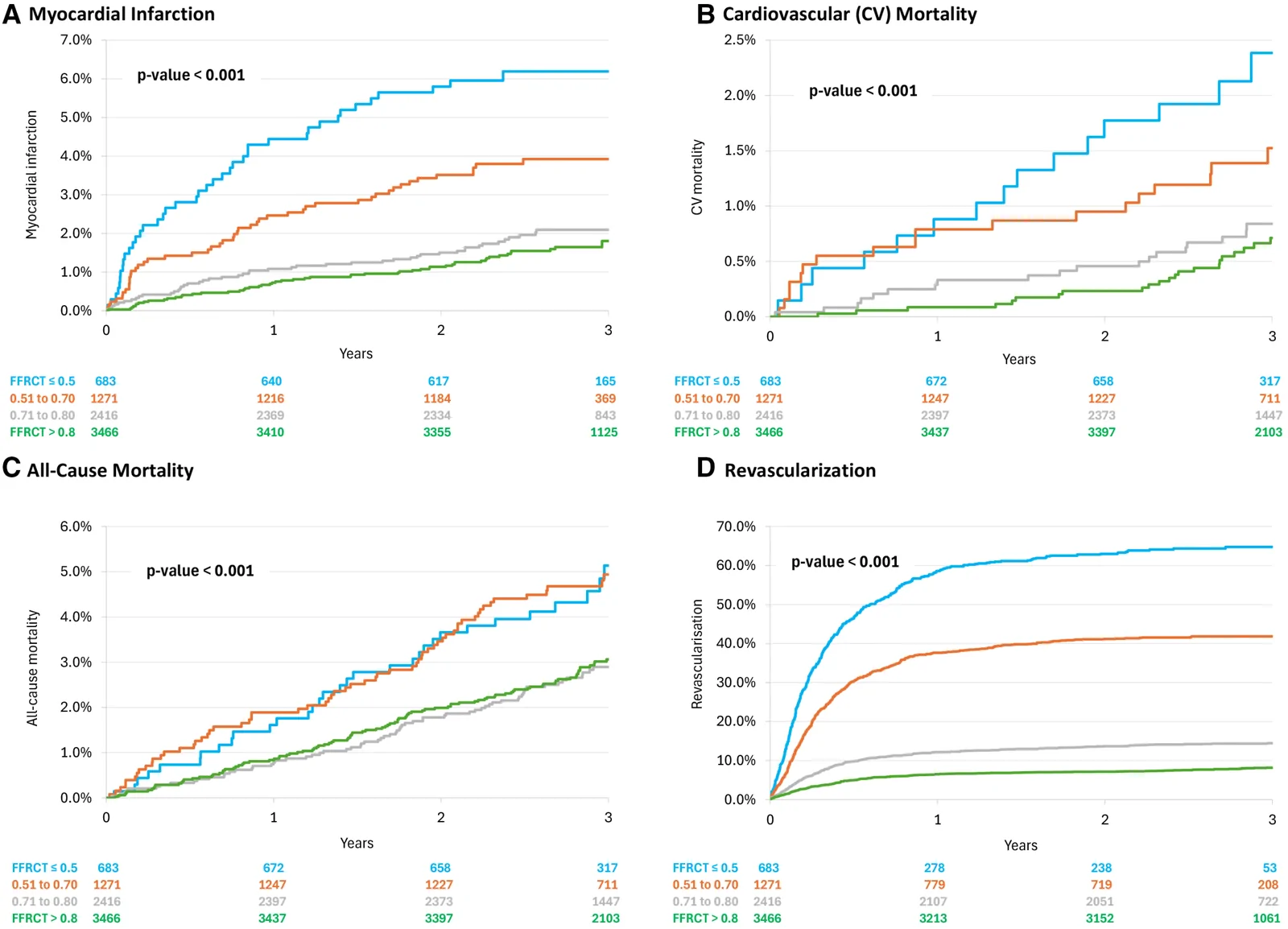
**Distal-vessel computed tomography angiography–derived fractional flow reserve (FFR-CT) subgroups and outcomes.** Shown are Kaplan-Meier curves for individual outcomes grouped according to distal-vessel FFR-CT values. Log-rank *P* values are shown.

### Comparison of Stenosis-Specific and Distal-Vessel FFR-CT

Models using stenosis-specific and distal-vessel FFR-CT categories were similar for discriminating individual cardiovascular outcomes. Stenosis-specific models had better fits for MI and revascularization, while distal models had a better fit for all-cause mortality (Table 2). When stenosis-specific FFR-CT was negative, cardiovascular event rates were low (1.4% at 3 years for nonfatal MI and cardiovascular death combined). In this group, distal FFR-CT values were concordant (ie, negative) in 63.4% of cases and discordant in 36.4% (35.1% borderline, 1.3% reduced, 0% severely reduced). Cardiovascular event rates were similar between concordant and borderline discordant groups (1.5% versus 1.3%, *P*=0.94) but higher when the distal FFR-CT was reduced (5.7%, *P*=0.004) (Figure 6).

**Figure 6.**
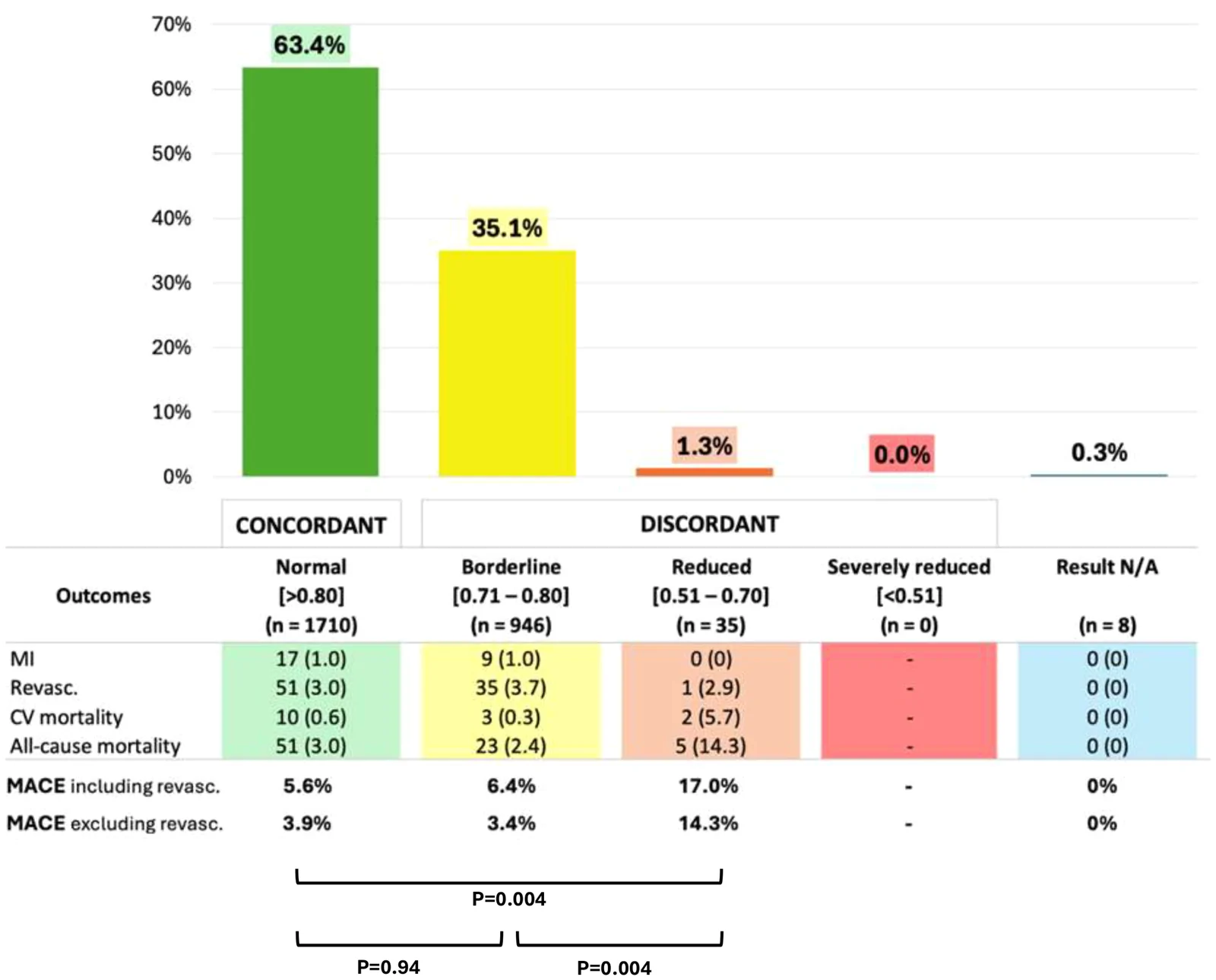
**Discordance analysis: clinical outcomes for distal-vessel computed tomography angiography–derived fractional flow reserve subgroups when stenosis-specific computed tomography angiography–derived fractional flow reserve is normal.** The bar chart illustrates the degree of concordance between distal and stenosis-specific computed tomography angiography–derived fractional flow reserve values when the stenosis-specific value is negative. The table illustrates the individual cardiovascular outcomes, cardiovascular event rates (nonfatal myocardial infarction [MI] and cardiovascular death) and major adverse cardiovascular event (MACE) rates (nonfatal MI, cardiovascular death, and revascularization [Revasc.]) for each distal-vessel subgroup. N/A indicates not available.

### Stenosis Location and Future Events

One hundred fifty-nine patients had distal stenoses in main epicardial vessels, as identified by a stenosis-specific FFR-CT being reported in the distal vessel. These patients had the highest rates of cardiovascular and all-cause mortality among all distal FFR-CT subgroups, with similar rates of MI and revascularization as the reduced distal FFR-CT subgroup (Figure S1). cardiovascular event rates (nonfatal MI or cardiovascular death) were similar regardless of the vessel system harboring the lowest stenosis-specific FFR-CT (left anterior descending artery, 3.1%, right coronary artery, 4.2%, left circumflex artery, 4.1%) (Table S4).

## DISCUSSION

This large multicenter study is the first to show that FFR-CT stratifies risk for the individual outcomes of MI, cardiovascular death, all-cause death, and revascularization at 3 years. Importantly, this is incremental to clinical risk factors and coronary stenosis severity. A risk continuum exists, as lower FFR-CT values are associated with incrementally worse prognosis, with an optimum stenosis-specific threshold of 0.67 for defining increased risk of future cardiac events. Stenosis-specific and distal-vessel FFR-CT models performed equivalently in discriminating outcomes, although stenosis-specific models had better fits for MI and revascularization.

Prior studies have pointed to the prognostic potential of FFR-CT in predicting cardiovascular events but have been limited by small numbers, composite outcomes, and short follow-up. The largest previous study, from the Danish ADVANCE registry, showed that a positive FFR-CT was associated with an increased risk of all-cause death and MI combined out to 7 years of follow-up.^17^ Our study reports a larger population with 10 times more clinical events, allowing us to determine that FFR-CT is associated with the individual outcomes of MI, cardiovascular death, and all-cause death independent of clinical risk factors and diameter of stenosis.

The incremental predictive capacity of adding FFR-CT to coronary stenosis adds to the debate around the prognostic relevance of myocardial ischemia compared with anatomical CAD severity. Post hoc analyses of large randomized intervention trials have demonstrated that anatomical disease burden may be more important than ischemia in predicting future major adverse cardiovascular events.^10,11^ In contrast, recent observational studies have suggested that ischemic burden provides independent and incremental prognostic value beyond CAD anatomy.^18,19^ None of these studies have examined the significance of vessel-specific anatomical disease burden and flow limitation combined. In our study, adding FFR-CT to CT-defined stenosis severity did improve the prediction of nonfatal MI or cardiovascular death. Interestingly, delta FFR-CT, the translesional gradient across a stenosis, akin to pull-back pressure gradient on invasive FFR, did not further improve model performance. Delta FFR-CT has been shown to improve identification of ischemic lesions and be the strongest predictor of future cardiac events, but these studies did not control for clinical risk factors.^20,21^

There is a gradient of risk, as lower FFR-CT values increase the likelihood of all of the individual outcomes. Clinical guidelines recommend a binary cutoff point at ≤0.80 for distinguishing normal from abnormal. This approach neglects important information about patient risk of future adverse events, which may be useful to inform patient-doctor decisions concerning the risk-benefit of revascularization but also the intensity of medical management. The association of FFR-CT subgroups and future events was strongest for MI, where all positive stenosis-specific FFR-CT subgroups (≤0.80) were associated with MI, with subgroups ≤0.70 associated with around 3 times increased MI risk, even when controlling for cardiovascular risk factors and diameter stenosis. The effect was less pronounced for mortality. While the reduced and severely reduced subgroups predicted cardiovascular and all-cause death on unadjusted analysis, only severely reduced FFR-CT predicted cardiovascular death in adjusted analyses. Importantly, a negative FFR-CT establishes patients in the same low-risk group as if they had minimal CAD, with cardiovascular event rates of approximately 1.5% at 3 years.

A borderline “gray zone” category has been established in invasive FFR and FFR-CT, recognizing lower comparative diagnostic accuracy, measurement variabilities, and lower evidence of treatment benefit.^22–24^ The exact range of the “gray zone” and how to interpret this category for patient management are frequently debated. Our data show that a borderline stenosis-specific FFR-CT (0.71–0.80) was associated with increased risk of future MI and revascularization but not survival. FFR-CT≤0.70 is associated with increased risk for cardiovascular and all-cause death, identifying those at highest risk for whom treatment may have the greatest potential to impact clinical outcomes. Borderline distal values, however, had no association with future cardiovascular events other than revascularization. Interpreting FFR-CT as a continuum for risk of future cardiac events would seem most appropriate. However, we did identify a stenosis-specific FFR-CT cutoff at 0.67 for the prediction of nonfatal MI or cardiovascular death. This value is lower than the diagnostic cutoff of ≤0.80 but does correspond to a previously reported invasive FFR cutoff of 0.67.^25^ It is consistent with the randomized placebo-controlled ORBITA-FIRE study, which showed that individualized invasive FFR thresholds for angina symptoms are considerably lower than the conventional ischemia-derived thresholds used to guide revascularization.^26^ The ≤0.80 threshold was defined following trials that included urgent or unplanned revascularization as an end point, where we excluded revascularization from the adverse cardiovascular outcome, focusing on MI and cardiovascular death.

Our observations raise the question as to whether distal vessel FFR-CT values should be reported, as the models based on distal-vessel and stenosis-specific FFR-CT perform similarly. The risk of a “don’t report distal” approach is that potentially important patient-specific information is not used, as reduced and severely reduced distal-vessel values do have an association with future cardiovascular events and all-cause death. This is corroborated by invasive studies showing that distal FFR in the 3 main epicardial vessels predicts major adverse cardiovascular events.^27^ Distal values may reflect plaque burden over the entire epicardial coronary artery rather than specific obstructive stenoses, with lower values suggesting diffuse disease and worse vascular health. Indeed, the plaque literature highlights the importance of total plaque volume across the coronary tree in predicting major adverse cardiovascular events, not just stenosis severity or presence of ischemia.^28^ However, it is challenging to disentangle the impact of distal FFR-CT from an upstream stenosis-specific FFR-CT, and here, discordance analyses may offer insight. In the instance of a negative stenosis-specific FFR-CT (>0.80) there was a positive distal FFR-CT in 36.4% of cases, but only 1.3% were reduced (≤0.7), with the rest borderline (0.71–0.80). The borderline group had the same cardiovascular event rates as the normal group, but cardiovascular event rates were significantly higher in the small group of patients with reduced distal values. Borderline distal values can occur in the absence of upstream plaque due to other mechanisms such as myocardial bridging, natural vessel tapering, and insufficient spatial resolution in small vessels.^29^ This may partly explain the absence of incremental prognostic benefit. Thus, it is reasonable to use stenosis-specific values alone for guiding the diagnosis, prognosis, and management strategy when the stenosis FFR-CT is positive. When there is distal and stenosis-specific discordance, in a small number of patients, distal FFR-CT provides potentially useful prognostic information when reduced ≤0.70. Interestingly, a subgroup of patients with distal stenoses in the main epicardial vessels had the highest rates of cardiovascular and all-cause mortality, with similar rates of MI and revascularization as the reduced FFR-CT group. This is a hypothesis-generating finding that raises questions around the dogma that only proximal disease is “prognostic.”^30,31^

### Limitations

As an observational study, the FFR-CT results likely influenced downstream patient management, especially revascularization. Despite this, we observe associations between FFR-CT and clinical outcomes. MI inherently spans several entities, including early events before revascularization, procedure-related events, and late events. Distinguishing periprocedural (type 4) events from post revascularization events that occurred during the same admission is not possible with our dataset. Data were not available for plaque burden, smoking status, and family history, which may be relevant for predicting cardiovascular outcomes. It has yet to be determined whether FFR-CT offers incremental prognostic value over total plaque burden.^32,33^ Future work should aim to integrate FFR-CT and clinical risk factors alongside other CCTA markers of risk, such as quantified plaque volumes and pericoronary fat attenuation, to develop improved personalized risk models for patients to guide preventative care.^28,34^

### Conclusion

FFR-CT predicts the clinical outcomes of MI, cardiovascular death, all-cause death, and revascularization independent of patient risk factors and diameter stenosis, with a gradient of risk where reduced and severely reduced FFR-CT values are linked to worse survival. Stenosis-specific and distal FFR-CT have similar discrimination for future events, although stenosis-specific models may be superior in predicting MI and revascularization. Discordance between stenosis-specific and distal FFR-CT is common, but in most cases, it would be reasonable to focus on stenosis-specific FFR-CT values for both diagnosis and prognosis of flow-limiting CAD.

## ARTICLE INFORMATION

### Acknowledgments

Liverpool Heart and Chest Hospital was the study sponsor and controller of the data. A Medical Research Council industrial collaboration agreement was signed between the sponsor and HeartFlow Inc. HeartFlow performed the FFR-CT analysis but did not fund the study, nor were they involved in analysis or interpretation of the data.

### Sources of Funding

### Disclosures

Dr Rogers and Dr Mullen are employees of HeartFlow. Dr Leipsic has share options and offers consultancy with Heartflow.

### Supplemental Material

Tables S1–S4

Figure S1

## Supplementary Material

## Appendix

FISH&CHIPS investigators for each site are as follows: Ashford and St Peters Hospital NHS Foundation Trust: Sumeet Sharma, Blackpool Teaching Hospitals NHS Foundation Trusts: Anoop Chauhan, Chee Liew, Dudley Group NHS Foundation Trust: Sawan Waidyanatha, Frimley Health NHS Foundation Trust: Sri Iyenger, Great Western Hospitals NHS Foundation Trust: Andrew Beale, Guys and St Thomas’ NHS Trust: Edward Nicol, Hull University Teaching Hospitals NHS Trust: Imran Sunderji, Leeds Teaching Hospitals NHS Trust: John Greenwood, Liverpool Heart and Chest Hospital NHS Foundation Trust: Timothy Fairbairn, Manchester University NHS Foundation Trust: Manish Motwani, Anna Reid, Matthias Schmitt, Newcastle Upon Tyne Hospitals NHS Foundation Trust: Anna Beattie, North Tees and Hartlepool NHS Foundation Trust: Justin Carter, Portsmouth Hospitals NHS Trust: Peter Haworth, Royal Devon and Exeter Hospital NHS Trust: Nicholas Bellenger, Royal Papworth Hospital NHS Foundation Trust: Jonathan Weir-McCall, Royal United Hospitals Bath NHS Foundation Trust: Benjamin Hudson, Jonathan Rodrigues, Sheffield Teaching Hospitals NHS Foundation Trust: Oliver Watson, United Lincolnshire Hospitals NHS Trust: Vinod Venugopal, University Hospitals Bristol NHS Foundation Trust: Mark Hamilton, University Hospital Dorset NHS Trust: Russell Bull, Peter O’Kane, University Hospitals of Leicester NHS Trust: Aparna Deshpande, Gerald McCann, University Hospitals of North Midlands NHS Trust: Simon Duckett, University Hospitals Sussex NHS Foundation Trust: Hatef Mansoubi, Victoria Parish, West Hertfordshire Hospital NHS Trust: Joban Sehmi
