# Supplementary material for: Prognostic Value of Coronary Computed Tomography Angiography–Derived Fractional Flow Reserve From a Large Nationwide Cohort
Source: Circulation. 2026 Sep 22;154(16):1580–91. doi: 10.1161/CIRCULATIONAHA.126.081264 (PMC13674600; doi:10.1161/CIRCULATIONAHA.126.081264)
Supplement: Supplementary file 1 [file cir-154-1580-s001.pdf]

Prognostic value of coronary computed tomography angiography-derived fractional flow reserve (FFR-CT) from a large nationwide cohort.

Supplementary materials.

Supplementary Table 1: Unadjusted Cox regression for association between FFR-CT subgroups and cardiovascular (CV) outcomes

Stenosis-specific FFR-CT								
	MI		CV mortality		All-cause mortality		Revascularization	
FFR-CT	HR (95% CI)	p	HR (95% CI)	p	HR (95% CI)	p	HR (95% CI)	p
> 0.80	Referent category	-	Referent category	-	Referent category	-	Referent category	-
0.71 to 0.80	2.08 (1.24-3.51)	0.006	1.51 (0.72-3.18)	0.28	1.01 (0.70-1.46)	0.94	4.79 (3.74-6.13)	<0.001
0.51 to 0.70	4.23 (2.66-6.73)	<0.001	2.05 (1.01-4.14)	0.046	1.44 (1.03-2.02)	0.03	13.04 (10.38-16.38)	<0.001
≤ 0.50	5.73 (3.66-8.97)	<0.001	3.40 (1.79-6.45)	<0.001	1.58 (1.13-2.21)	0.01	24.48 (19.60-30.57)	<0.001
N/A	0.69 (0.26-1.79)	0.44	1.19 (0.43-3.27)	0.74	0.63 (0.36-1.12)	0.11	0.33 (0.16-0.67)	0.002
AIC	3310.07		1307.22		4641.64		25850.80	
**Distal vessel FFR-CT**								
	MI		CV mortality		All-cause mortality		Revascularization	
FFR-CT	HR (95% CI)	p	HR (95% CI)	p	HR (95% CI)	p	HR (95% CI)	p
> 0.80	Referent category	-	Referent category	-	Referent category	-	Referent category	-
0.71 to 0.80	1.23 (0.83-1.82)	0.29	1.24 (0.67-2.30)	0.49	0.95 (0.70-1.30)	0.76	1.89 (1.62-2.22)	<0.001
0.51 to 0.70	2.46 (1.67-3.62)	<0.001	2.29 (1.23-4.28)	0.009	1.68 (1.22-2.31)	0.001	6.67 (5.76-7.73)	<0.001
≤ 0.50	4.05 (2.70-6.06)	<0.001	3.63 (1.88-7.00)	<0.001	1.66 (1.12-2.48)	0.01	12.52 (10.75-14.59)	<0.001
AIC	3352.51		1304.74		4640.47		26434.35	

Abbreviations: Akaike information criterion (AIC), cardiovascular (CV), myocardial infarction (MI)

Supplementary Table 2: Sensitivity analysis with cases with occluded vessels excluded. Multivariable Cox regression for association between FFR-CT subgroups and cardiovascular (CV) outcomes, adjusted for age, sex, hypertension, diabetes, dyslipidaemia and CAD-RADS category.

Stenosis-specific FFR-CT								
	MI		CV mortality		All-cause mortality		Revascularization	
FFR-CT	HR (95% CI)	p	HR (95% CI)	p	HR (95% CI)	p	HR (95% CI)	p
> 0.80	Referent category	-	Referent category	-	Referent category	-	Referent category	-
0.71 to 0.80	1.52 (0.86-2.70)	0.15	1.41 (0.60-3.28)	0.43	0.99 (0.66-1.46)	0.95	3.01 (2.30-3.93)	<0.001
0.51 to 0.70	2.88 (1.70-4.89)	<0.001	2.27 (0.99-5.22)	0.053	1.40 (0.95-2.07)	0.09	6.29 (4.89-8.08)	<0.001
≤ 0.50	3.37 (1.92-5.92)	<0.001	2.73 (1.08-6.88)	0.03	1.28 (0.80-2.05)	0.30	9.29 (7.19-12.00)	<0.001
N/A	1.00 (0.34-2.95)	>0.99	1.18 (0.37-3.77)	0.78	0.72 (0.38-1.34)	0.30	0.70 (0.32-1.52)	0.36
AIC	2569.60		912.32		3627.38		18589.27	
**Distal vessel FFR-CT**								
	MI		CV mortality		All-cause mortality		Revascularization	
FFR-CT	HR (95% CI)	p	HR (95% CI)	p	HR (95% CI)	p	HR (95% CI)	p
> 0.80	Referent category	-	Referent category	-	Referent category	-	Referent category	-
0.71 to 0.80	0.94 (0.60-1.45)	0.77	0.86 (0.39-1.88)	0.70	0.78 (0.55-1.10)	0.16	1.26 (1.05-1.51)	0.02
0.51 to 0.70	1.38 (0.87-2.18)	0.17	2.78 (1.29-5.96)	0.009	1.61 (1.11-2.34)	0.01	2.96 (2.48-3.53)	<0.001
≤ 0.50	2.11 (1.27-3.51)	0.004	4.01 (1.61-10.00)	0.003	1.17 (0.68-2.01)	0.57	4.33 (3.58-5.25)	<0.001
AIC	2582.00		901.46		3618.00		18741.54	

Abbreviations: Akaike information criterion (AIC), cardiovascular (CV), confidence interval (CI), hazard ratio (HR), myocardial infarction (MI)

Supplementary Table 3. Multivariable Cox regression for association between the mean distal FFR-CT value across all 3 epicardial coronary arteries, adjusted for age, sex, hypertension, diabetes, dyslipidaemia and CAD-RADS category

Mean distal FFR-CT	MI		CV death		All-cause death		Revascularization	
	HR (95% CI)	p	HR (95% CI)	p	HR (95% CI)	p	HR (95% CI)	p
> 0.90 (n=1026)	Reference		Reference		Reference		Reference	
0.81 to 0.90 (n=4506)	0.90 (0.52, 1.56)	0.71	0.94 (0.41, 2.17)	0.89	0.85 (0.56, 1.28)	0.44	1.06 (0.83, 1.36)	0.65
0.71 to 0.80 (n=1482)	1.19 (0.66, 2.12)	0.56	1.19 (0.45, 3.12)	0.72	1.19 (0.74, 1.92)	0.47	2.58 (2.02, 3.31)	<0.001
0.61 to 0.70 (n=460)	1.60 (0.83, 3.09)	0.16	0.97 (0.27, 3.53)	0.97	1.01 (0.53, 1.95)	0.97	3.23 (2.47, 4.22)	<0.001
0.51 to 0.60 (n=130)	1.20 (0.46, 3.15)	0.71	2.33 (0.55, 9.86)	0.25	2.77 (1.32, 5.82)	0.007	3.31 (2.38, 4.60)	<0.001
≤ 0.50 (n=71)	0.89 (0.20, 3.92)	0.88	4.72 (1.14, 19.61)	0.03	2.72 (1.10, 6.76)	0.03	2.99 (1.97, 4.55)	<0.001

Supplementary Table 4. Cardiovascular outcomes based on the vessel system harbouring the lowest stenosis-specific FFR-CT value

	**LAD** (n = 4350)	**RCA** (n = 1142)	**LCx** (n = 1592)	**No result** (n = 752)
**MI**	102 (2.3)	36 (3.2)	48 (3.0)	5 (0.7)
**CV death**	40 (0.9)	11 (1.0)	1.1 (1.1)	5 (0.7)
**All-cause death**	154 (3.5)	38 (3.3)	55 (3.5)	14 (1.9)
**Revascularization**	1003 (23.1)	267 (23.4)	295 (18.5)	8 (1.1)

Abbreviations: cardiovascular (CV), left anterior descending artery (LAD), left circumflex artery (LCx), myocardial infarction (MI), right coronary artery (RCA)

Supplementary figure 1: Distal vessel FFR-CT subgroups and outcomes without redistribution of 'no distal pin' subgroup

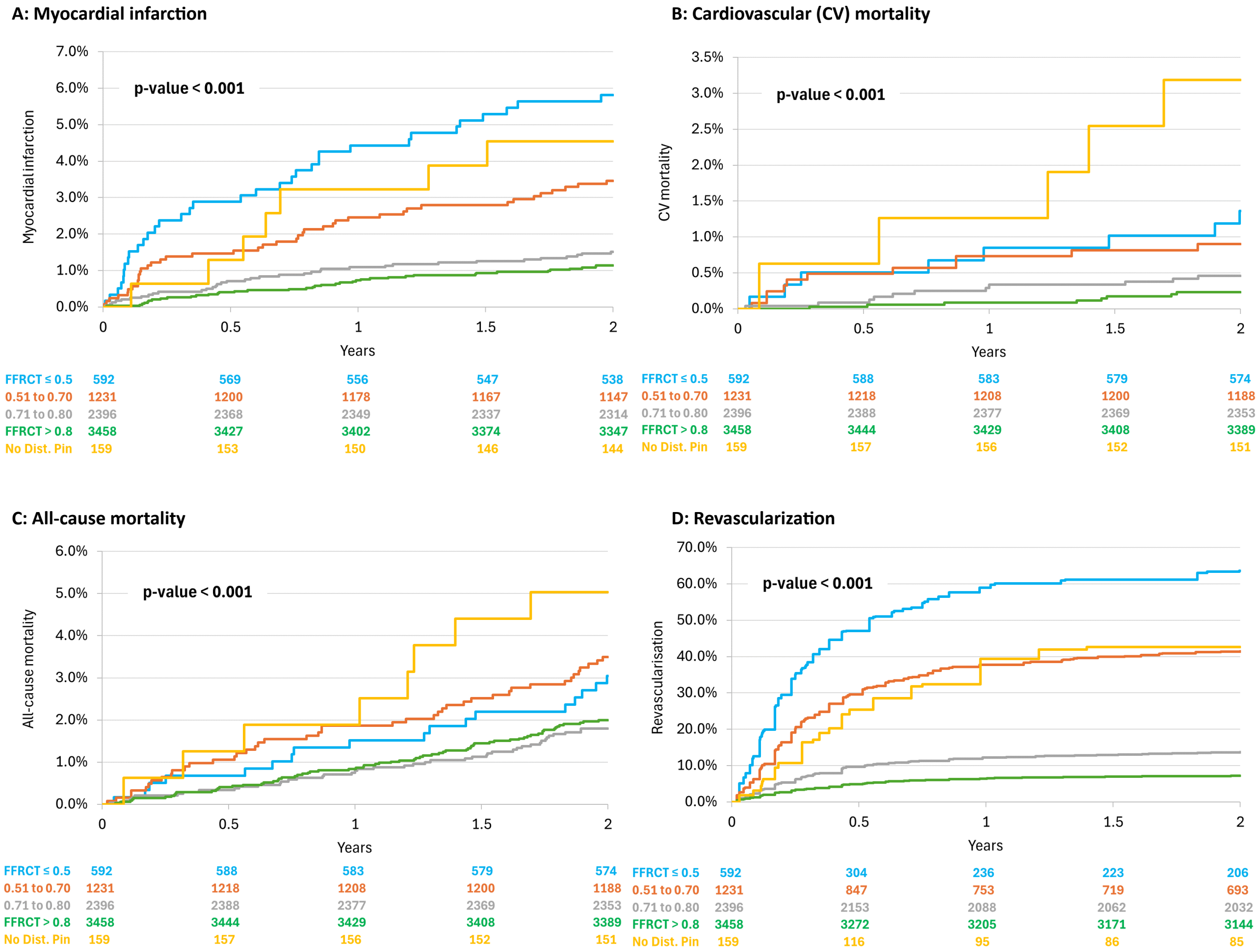

Kaplan-Meier curves for cardiovascular outcomes grouped according to distal vessel FFR-CT. For 'no distal pin' there was a stenosis-specific FFR-CT value in the distal vessel so a further distal FFR-CT value was not reported.
